# A cross-sectional study of early marriage among ever-married Somali women: Prevalence, regional differences, and sociodemographic determinants

**DOI:** 10.1371/journal.pone.0329166

**Published:** 2025-07-28

**Authors:** Abdirahman Saeed Mohamed

**Affiliations:** School of Graduate Studies University of Hargeisa, Hargeisa, Somaliland; Higher Education Partnership / Erasmus University Rotterdam, ETHIOPIA

## Abstract

This study aims to examine the prevalence of early marriage among ever-married Somali women aged 20−49, assess differences across age groups and regions, and identify the main sociodemographic and contextual determinants. The study used the 2020 Somali Health and Demographic Survey dataset. The survey was a representative, three-stage cluster stratified sample, and the data were collected through personal interviews. Descriptive statistics and chi-square tests were used to examine prevalence and differences by background characteristics. Logistic regression modeled the probability of women marrying before 15 or 18 while adjusting for sociodemographic and contextual variables. Three logistic regression models were run; two of these models examined women aged 20−49 for the probability of marrying below age 15 or age 18, while one considered women aged 20−29 in assessing the probability of marriage before age 18. Of the respondents, 24.3% (95% CI: 22.6–26.1) married before 15, while the overall estimated prevalence of marriage before 18 was 41.7% (95% CI: 39.3–44.2). The odds of getting married before 15 (AOR = 1.51, 95% CI: 1.16–1.99, p = 0.002) and 18 (AOR = 2.08, 95% CI: 1.68–2.58, p < 0.001) were higher for the younger age group, 20–29. Women from Somaliland showed lower odds for marrying before age 15 (AOR = 0.50, 95% CI: 0.41–0.62, p < 0.001) and below age 18 (AOR = 0.56, 95% CI: 0.46–0.66, p < 0.001). Women from lower socioeconomic status showed higher probabilities of early marriage. In the models for women aged 20–49, education had no significant effect. Only among women aged 20–29, education was unexpectedly linked to early marriage, and there were no differences by living in an urban or rural area. Odds ratios were higher for those who accepted domestic violence and lower for those who ever used the internet and participated in household decision-making.

## Introduction

Early or child marriage is defined as marriage before the age of 18 [1–3]. It is both common and entrenched across the world [4] particularly in the developing world. Early marriage has serious negative effects on individuals’ social, financial, and health aspects, affecting families, communities, and individuals alike [2]. It impacts the lives of girls more than boys [2]; girls who marry early have more children throughout their reproductive lifespan than those who marry after the age of 18, which has negative consequences on the health and well-being of both girls and their infants [5,6]. Social norms, economic pressure, gender inequality, and insecurity are main reasons for child marriage for girls [7–12]. In Africa, the main predictors of early marriage for women who have already been married are socioeconomic status, employment status, urban/rural residence, and educational attainment [13]. In the present study, the terms child marriage and early marriage are used interchangeably to refer to marriage before the age of 18, in line with the existing literature [1–3].

Somalia has endured endless conflict, perpetual political instability, and catastrophic natural disasters for decades, resulting in a chronic humanitarian emergency. Forced displacement has spread into many communities across the country, touching the lives of numerous Somalis, who are displaced, either internally or to neighboring countries as refugees [14]. This further increases the risks faced by the displaced communities, most especially children, women, and vulnerable individuals. Such groups are now more likely to fall victim to abuse, discrimination, and violence, including GBV and trafficking [15,16].

Somalia is one of the countries with the lowest levels of human development and gender inequality [17,18]. However, gender-based violence, female genital cutting/mutilation (FGM/C), and harmful traditions persist, which make it difficult for Somalia as it struggles through chronic insecurity and humanitarian crises [19]. The Somali patriarchal culture, reinforced by religion, governs marriage and gender relations in Somalia [20]. However, the absence of a civil registration system, inadequate legal framework, and enforcement mechanisms, and customary laws condone the practice and are the driving factors of early marriage in Somalia [21]. As per Article 28.5 of the 2012 Somali Constitution, a marriage cannot be regarded as lawful unless both the man and the woman willingly consent to it, and if one of them has not reached the age of maturity [22]. The age of maturity isn’t stated in the Constitution, and the date of birth is not a determining factor for adulthood. Instead, the majority of Somalis rely on Sharia and traditional laws, where reaching puberty serves as the criterion for adulthood [23–25]. Traditionally, Somali boys can marry around their seventeens or eighteens while girls enter their first marriage at 15, and some may already be widowed or divorced [26]. The Somali Family Code of 1975 states that the legal age for marriage is eighteen for both sexes, yet females as young as sixteen or seventeen may marry with permission from their parents or guardians [27]. This indicates that Somalia’s legal framework fails to protect against early marriage and that there is a significant inconsistency in the application of the laws. In Somaliland, although not directly referring to child marriage, the constitution entrenches the values of equality and non-discrimination under Article 8 and protective provisions outlined under Article 19, which together seek to protect the vulnerable in society [28]. These provisions are particularly relevant in issues around marriage and rights therein.

Early marriage is still a widespread and acceptable practice in Somali society, rooted in cultural norms and beliefs [18], usually triggered due to economic and cultural factors, where parents can marry their daughters early due to poverty, cultural beliefs, or to mitigate economic burden. It denies the girl an education and opportunities and perpetuates the vicious circles of poverty and inequality based on gender [29]. However, a recent study established a new relationship between the social norms and agency regarding adolescent marriage [30]. The study shows that while adolescent girls might think they have the agency to make decisions about marriage, their decisions are considerably shaped by the social norms in the environment. Most adolescent girls who are wed believe they have a level of agency for these decisions. However, within this perceived agency, the fundamental social norms based on gender, family, and culture considerably limit the choices of adolescent girls as they consider marriage [30].

Despite improvements in security and humanitarian situation, early marriage remains a significant issue in Somalia, as many girls marry before 18 [20]. However, Somalia is challenged by large data gaps across its different sectors, mainly stemming from continued insecurities and instability. The most recent population census in Somalia was in 1985–1986, yet its findings remained undisclosed to the public [31]. Subsequently, due to Somalia’s instability over the past thirty years, conducting comprehensive data collection endeavors has been unfeasible. There’s also a scarcity of systematic investigations focusing on early marriage and marriage patterns in Somalia.

This study examines early marriage among ever-married women in Somalia and Somaliland. Somaliland declared its independence in 1991 from Somalia as a result of the collapse of the central government of Somalia. Though yet to gain international recognition, Somaliland enjoys relative political stability and security in contrast to the situation in Somalia [32,33]. The study used the Somali Health and Demographic Survey 2020 (SHDS 2020), which is a representative sample survey carried out in Somalia and Somaliland. The analyses focused on ever-married women aged 20–49 years. These women passed the threshold of 18 years, which is an internationally recognized cutoff of early marriage [1–3], hence allowing for accurate retrospective analysis of early marriage and related factors. The study aims to:

1. Determine the prevalence of early marriage among ever-married women aged 20–49 years, differences across regions, and age groups.
2. Examine the sociodemographic determinants and contextual factors underlying early marriage for Somali women.

## Methods and materials

### Data

The research was based on data collected through the 2020 Somali Health and Demographic Survey (SHDS 2020). This large-scale survey used a rigorous sample design that covered a range of dimensions, ranging from housing and child health to nutrition, fertility, family planning, female circumcision, gender-based violence, chronic conditions, and anything related to knowledge, attitudes, and perceptions about HIV/AIDS. Using a three-stage cluster stratified sample, the research used probability proportional to size sampling for the primary and secondary sample units, coupled with systematic sampling for the third. Trained enumerators entered into 15,870 households using structured questionnaires administered through personal interviews, and collected data through individual interviews for 16,715 women between 15 and 49 years old [34]. The data used in this study are available from the Somali National Bureau of Statistics microdata repository. Access to the data is free but requires free registration and approval from the data custodian. Also, the dataset contained no personally identifiable information, guaranteeing confidentiality and anonymity. The dataset is available at: https://microdata.nbs.gov.so/index.php/catalog/50

### Sample

The present study included 10337 ever-married women aged 20–49 who had complete data on age at first marriage. These women were drawn from the women’s record file (SOIR), which included 16,486 women of reproductive age, and was used to extract all variables in the study. Analyses were confined to the age group 20–49 to ensure that all women had passed the age threshold for early marriage (18 years old), allowing for an accurate estimation of prevalence and determinants. Since the study participants were adult women aged 20–49, the term ‘women’ was used throughout the study. The term ‘girls’ was used where appropriate.

### Measurement

#### Dependent variable.

In the 2020 SHDS, women were asked to report the month and year of their first marriage. The age at first marriage was then calculated by subtracting the date of birth from the date of the first marriage. In this study, early marriage was operationalized as a binary variable in which “0” was coded as marriage at 18 or older, and “1” for marriage before 18. This final binary variable was used in the analysis.

#### Sociodemographic determinants.

Women’s ages were grouped into three cohorts: 20–29, 30–39, and 40–49 [35]. These age groups help examine the differences in the likelihood of early marriage among different age cohorts. Educational attainment was ascertained through questions regarding the highest level and grade completed, resulting in ordinal categories of no education, primary, secondary, and higher. Due to low frequencies in secondary and higher categories, which could lead to unstable estimates, the variable was dichotomized into “no education” and “primary or higher” by combining the latter three categories. Employment status was determined by whether the respondent was working at the time of the survey, with “yes” and “no” responses. Internet usage was assessed based on a lifetime use question with the response categories “yes” and “no”. In the 2020 SHDS, household socioeconomic status was measured using a wealth quintile index based on household asset data through principal component analysis [35]. The index groups households into five groups (lowest, second, middle, fourth, and highest). In the present study, to simplify analysis and avoid sparse categories in the regression analysis, the categories were regrouped into (lowest, middle, and highest) by combining the lowest and second, and the fourth and middle.

The original three-level residence type (“urban”, “rural”, and “nomadic”), was transformed into two categories by grouping “rural” with “nomadic” due to their similar characteristics. In the 2020 SHDS, stratification was based on the 18 administrative regions of Somalia predating the civil war in 1988, in conjunction with residence type. To ensure comparability, the region variable was re-categorized into “Somaliland” and “Somalia”, with the latter encompassing the Puntland state of Somalia and the South-Central Somalia regions (13 regions in total). Somaliland represents the other five regions.

#### Contextual variables.

Examining early marriage practices requires understanding cultural norms surrounding gender roles and relationships. The 2020 SHDS didn’t include data on social norms and culture. However, attitudes towards intimate partner violence (IPV) are a useful surrogate measure of these norms. In the context of strongly held patriarchal values within Somali culture, it is plausible that attitudes toward intimate partner violence (IPV) mirror a broader cultural attitude toward male authority and dominance in relationships. To explore this, women were surveyed about their attitudes toward IPV using a six-item scale. They were asked their opinion on whether a husband is justified in hitting or beating his wife, “if she wastes resources, if she refuses to have sex with him, if she argues with him, if she neglects household duties, if she neglects the children, and if she goes out without telling him.” A binary variable was derived from the six-item scale with the response categories (Doesn’t justify IPV = 0, justifies IPV for at least one reason = 1). The study predicts that women who justify IPV are more likely to marry early compared to those who do not justify IPV.

Religious beliefs, in particular views on Female Genital Mutilation/Cutting (FGM/C), provide another perspective for analyzing gender and sexuality related cultural norms. In the Somali society, FGM/C is deeply rooted in tradition and cultural beliefs and reflects purity and obedience. Religious beliefs of FGM entwine with cultural customs, making it a salient proxy indicator of cultural norms about gender and sexuality. Women’s religious beliefs on FGM/C were measured by the question “Do you believe that female circumcision is required by your religion?” With the response categories “yes” or “no”. The prediction is that women who believe FGM/C is required by religion might marry before 18 compared to those who do not hold this belief. Finally, examining the women’s participation in household decision-making enables to uncovering of underlying gender dynamics and power relations within families. This is relevant in societies where gender roles may influence decision-making about marriage, including the age at which women marry. In the 2020 SHDS, women’s participation in household decision-making was measured by asking these four items: 1. “Person who usually decides on large household purchases”, 2 “Person who usually decides on respondent’s health care”, 3 “Person who usually decides on visits to family or relatives”, and 4 “Person who usually decides what to do with money husband earns”. A binary variable reflecting women’s participation was derived from these four items (respondent decides or jointly decides with husband = 0, husband or other decides = 1), with the prediction that women who do not participate in household decision-making are more likely to marry early compared to those who do participate.

### Data quality

An adequate sample size is one of the fundamental requirements of research quality and the present study included a large sample of ever-married women to examine early marriage. To evaluate data quality, the completeness of reported age at first marriage was assessed, and inconsistencies and outliers were checked in the data. The age distribution reported was examined for indications of digit preference. To estimate the presence of age heaping, usually measured using Whipple’s index, values range from 100, which implies an absence of age heaping, to 500, which implies a high presence of age heaping. For the data at hand, Whipple’s index for the age at first marriage was 124, implying a mild presence of heaping, since an index between 125 and 174 indicates rough data quality [36,37].

### Statistical analyses

The data analysis and data management were conducted using the 2020 release of IBM SPSS Statistics for Windows, Version 27.0. Armonk, NY: IBM Corp. Missing data was handled using listwise deletion, also known as complete-case analysis. To present the background of the sample and determine the prevalence of early marriage among ever-married Somali women and its regional variations, descriptive statistics, percentages with 95% confidence intervals (CI), and chi-square tests were utilized. The descriptive statistics and 95% CI considered the women’s weighting variable and the complex sample design of the 2020 SHDS.

Multivariable logistic regression was used to examine the sociodemographic determinants and contextual influences contributing to early marriage among Somali women. This method enabled modeling the association between explanatory variables and the conditional probability of marrying before the age of 15 or 18 among ever-married Somali women. Three logistic regression models were run:

1. The first model was based on all women aged 20–49 with the conditional probability of marrying before 18. This model provides a comprehensive explanation of the sociodemographic and contextual factors associated with early marriage (before 18) across a broad age range of women.
2. The second model was based on women aged 20–49 with the conditional probability of marrying before 15. This model concentrates on marriages before 15, but in the same age group. It specifies the circumstances that are potentially most important in very early marriages.
3. The third model included women aged 20–29 with the conditional probability of marrying before 18. Younger women may have different social and economic experiences compared to older women. Concentrating on this younger age group can help identify more recent influences and trends that are less apparent in the wider age group.

The Neglect of the design features of a complex sample while analyzing the data could result in significantly biased conclusions [38,39]. To account for the complex sample design, like clustering and stratification effects, logistic regression was conducted using the IBM SPSS complex samples module [40]. The alpha level for the study was set at 5%. All variables were included in the model regardless of their statistical significance in the bivariate analysis. The selection of variables was based on theoretical and existing literature indicating that the included variables affect early marriage [1–3,13], and including all variables improves control for confounding. The explanatory variables were tested for multicollinearity by the Variance Inflation Factor (VIF) values calculated from an ordinary least squares (OLS) regression model [41]. VIF values ranged from 1.03 to 1.74, reflecting no serious multicollinearity.

## Results

### Descriptive results

The average age of the women was 31 years (SD = ±7.24). Approximately 84% had no education, and 91% were unemployed. The majority, 64%, were from rural areas. Regarding region, 63% were from Somalia, and 37% were from Somaliland. A significant proportion, belonging to the lowest socioeconomic status, 43% (see Table 1).

**Table 1:** Background characteristics of women (n = 10337).

	n	%	95% CI	
			LL	UL
**Age** mean = 31.06, SD = ±7.24				
20-29	4692	45.3	43.8	46.9
30-39	3900	37.8	36.4	39.2
40-49	1745	16.9	15.9	18.0
**Education**				
No education	8612	84.1	82.2	85.9
Primary or higher	1725	15.9	14.1	17.8
**Employment status**				
Yes	897	8.6	7.8	9.5
No	9425	91.4	90.5	92.2
**Socioeconomic status**				
Lowest	4181	42.7	38.4	47.1
Middle	4061	38.9	35.8	42.2
Highest	2095	18.4	16.1	20.9
**Type of residence**				
Rural	5593	63.8	59.4	67.9
Urban	4744	36.2	32.1	40.6
**Region**				
Somaliland	3990	37.2	30.2	44.8
Somalia	6347	62.8	55.2	69.8

Note:

1 SD = standard deviation, CI = Confidence Interval, LL = Lower limit, UL = Upper limit.

2 Percentages and confidence intervals consider complex sample design of SHDS.

3 Employment status had 15 missing cases.

### Prevalence of early marriage among ever-married Somali women

**Fig 1:**
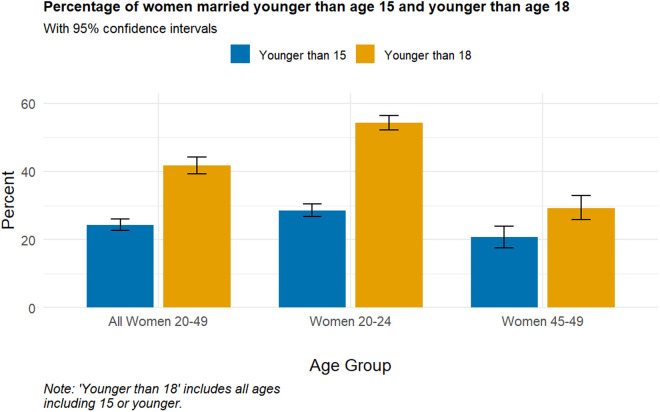
Prevalence of early marriage among ever-married Somali women (n = 10337).

The age at first marriage among ever-married women ranged as low as 10–47 years. The average age at first marriage was 19.08 years (SD = ±5.61), and the median age of 19 years. As indicated in Fig 1, the overall prevalence of marriage before the age of 18 among women aged 20–49 was 41.7% (95% CI: 39.3–44.2), while those who got married before age 15 accounted for 24.3% (95% CI: 22.6–26.1). In the age group 20–24, about 54.2% (95% CI: 50.0–58.3) married before 18, and 28.6% (95% CI: 25.9–31.5) married before age 15. However, for older women in the age group 45–49, the percentage who were married before age 18 was lower, around 29%, and those married before age 15 was approximately 21%.

### Prevalence of early marriage by background characteristics

Prevalence of early marriage differs in terms of age groups, socioeconomic status, and regions. A higher prevalence was reported by women from lower socioeconomic backgrounds and those from Somalia compared to Somaliland. The chi-square tests indicated that no statistical differences in prevalence according to education, employment status, or type of residence. However, contrary to expectations, early marriage was more prevalent among women with primary or higher education levels and those residing in urban areas (see Table 2).

**Table 2:** Early marriage and background factors: χ² results (n = 10,337).

	Age at first marriage <18				P-value (χ^2^)
	n	%	95% CI		
			LL	UL	
**Age**					
20-29	2384	48.1	44.8	51.4	<0.001 (159.01)
30-39	1585	38.2	35.6	40.8	
40-49	599	32.6	29.4	35.9	
**Education**					
No education	3777	41.4	38.7	44.1	0.242 (2.79)
Primary or higher	791	43.6	40.0	47.2	
**Employment status**					
Yes	394	40.1	35.6	44.8	0.452 (1.07)
No	4170	41.9	39.4	44.5	
**Socioeconomic status**					
Lowest	1979	41.9	38.5	45.4	<0.001 (70.86)
Middle	1866	45.3	41.5	49.2	
Highest	723	33.8	30.6	37.0	
**Type of residence**					
Rural	2526	41.0	37.6	44.4	0.401 (4.47)
Urban	2042	43.1	39.5	46.7	
**Region**					
Somaliland	1435	34.2	31.7	36.8	<0.001 (143.0)
Somalia	3133	46.2	42.8	49.6	

Note. CI = confidence interval, LL = lower limit, UL = upper limit.

Calculations consider the complex sample design of SHDS 2020.

### Logistic regression results

#### Model 1.

In Table 3, the results of Model 1 indicate that a number of factors are associated with the probability of marrying before 18 years old among ever-married Somali women aged 20−49. Compared to women in the age group 40−49, women aged 20−29 and 30−39 had higher adjusted odds of marrying before 18 (AOR = 2.08, 95% CI: 1.68–2.58, p < 0.001) and (AOR = 1.39, 95% CI: 1.13–1.73, p = 0.002), respectively. Women in the lowest and middle socioeconomic status were more likely to marry early compared to those in the highest status (AOR = 1.51, 95% CI: 1.17–1.94, p = 0.001) and (AOR = 1.41, 95% CI: 1.11–1.79, p = 0.005). Moreover, accepting IPV increased the odds of early marriage (AOR = 1.22, 95% CI: 1.02–1.47, p = 0.032), whereas involvement in household decision-making decreased the odds (AOR = 0.69, 95% CI: 0.58–0.81, p < 0.001). Women who have ever used the internet had a decreased odds ratio (AOR = 0.77, 95% CI: 0.61–0.98, p = 0.034). Regionally, women in Somaliland were less likely to experience early marriage as compared to Somalia (AOR = 0.56, 95% CI: 0.46–0.66, p < 0.001).

**Table 3:** Odds ratios for early marriage before 18 among women aged 20-49 (n = 6,058).

Model 1	OR	95% CI		AOR	95% CI		p-value
		LL	UL		LL	UL	
**Age**							
20-29	1.92	1.62	2.28	2.08	1.68	2.58	<0.001
30-39	1.28	1.09	1.51	1.39	1.13	1.73	0.002
40-49 (ref)							
**Education**							
No education	0.91	0.79	1.06	0.83	0.67	1.01	0.063
Primary or higher (ref)							
**Currently working**							
Yes	0.93	0.77	1.13	1.03	0.79	1.34	0.840
No (ref)							
**Ever used the internet**							
Yes	0.95	0.78	1.15	0.77	0.61	0.98	0.034
No (ref)							
**Socioeconomic status**							
Lowest	1.41	1.15	1.75	1.51	1.17	1.94	0.001
Middle	1.63	1.34	1.97	1.41	1.11	1.79	0.005
Highest (ref)							
**Justifies wife-beating**							
Yes	1.19	1.01	1.40	1.22	1.02	1.47	0.032
No (ref)							
**Believe FGM is required by religion**							
Yes	1.20	1.02	1.41	1.06	0.90	1.24	0.484
No (ref)							
**Participates in HH decision-making**							
Yes	0.63	0.54	0.73	0.69	0.59	0.81	<0.001
No (ref)							
**Type of residence**							
Rural	0.92	0.75	1.12	0.95	0.74	1.21	0.66
Urban (ref)							
**Region**							
Somaliland	0.61	0.51	0.72	0.56	0.46	0.66	<0.001
Somalia (ref)							

Note. OR = odds ratio, AOR = adjusted odds ratio, ref. = reference Category.

The results account for the 2020 SHDS complex sample design.

#### Model 2.

The results of Model 2 as shown in Table 4, indicate that women in the 20–29 and 30−39 age groups were also more likely to marry before the age of 15 with (AOR = 1.51, 95% CI: 1.16–1.99, p = 0.002) and (AOR = 1.37, 95% CI: 1.05–1.79, p = 0.022), respectively. Women with a low socioeconomic background showed a higher likelihood of getting married before 15. Yet, women who were involved in household decision-making had a lower odds ratio of marrying before 15 (AOR = 0.73, 95% CI: 0.58–0.92, p = 0.005). As in Model 1, women from Somaliland were also less likely to marry before 15 compared to those from Somalia (AOR = 0.50, 95% CI: 0.41–0.62, p < 0.001).

**Table 4:** Odds Ratios for early marriage before 15 among women aged 20–49 (n = 6,058).

Model 2	OR	95% CI		AOR	95% CI		p-value
		LL	UL		LL	UL	
**Age**							
20-29	1.39	1.14	1.70	1.51	1.16	1.99	0.002
30-39	1.22	1.02	1.47	1.37	1.05	1.79	0.022
40-49 (ref)							
**Education**							
No education	1.19	0.99	1.43	1.06	0.84	1.34	0.631
Primary or higher (ref)							
**Currently working**							
Yes	0.95	0.76	1.20	1.19	0.83	1.71	0.355
No (ref)							
**Ever used the internet**							
Yes	0.82	0.68	0.99	0.82	0.55	1.22	0.324
No (ref)							
**Socioeconomic status**							
Lowest	1.64	1.36	1.96	1.53	1.17	2.00	0.002
Middle	1.79	1.51	2.11	1.41	1.10	1.79	0.006
Highest (ref)							
**Justifies wife-beating**							
Yes	1.18	0.99	1.41	1.18	0.97	1.44	0.101
No (ref)							
**Believe FGM is required by religion**							
Yes	1.29	1.10	1.51	1.18	0.98	1.41	0.085
No (ref)							
**Participates in HH decision-making**							
Yes	0.61	0.52	0.71	0.69	0.58	0.82	0.000
No (ref)							
**Type of residence**							
Rural	0.97	0.83	1.14	0.90	0.71	1.15	0.400
Urban (ref)							
**Region**							
Somaliland	0.59	0.51	0.69	0.50	0.41	0.62	0.000
Somalia (ref)							

Note. OR = odds ratio, AOR = adjusted odds ratio, ref. = reference Category.

The results account for the 2020 SHDS complex sample design.

#### Model 3.

The results of Model 3 are presented in Table 5. Compared to women with primary or higher educational level, women who had no education had decreased odds of being married before (AOR = 0.71, 95% CI: 0.55–0.91, p = 0.006). This result contrasts with the literature. However, women who were working had reduced odds of early marriage (AOR = 0.58, 95% CI: 0.34–0.96, p = 0.035). Women who involved in household decision-making once again had lower odds of early marriage (AOR = 0.73, 95% CI: 0.55–0.94, p = 0.016), and women from Somaliland continued to have lower odds of early marriage compared to those from Somalia (AOR = 0.52, 95% CI: 0.41–0.66, p < 0.001).

**Table 5:** Odds ratios for early marriage before 18 among women aged 20–29 (n = 2,861).

Model 3	OR	95% CI		AOR	95% CI		p-value
		LL	UL		LL	UL	
**Education**							
No education	0.82	0.67	0.99	0.71	0.55	0.91	0.006
Primary or higher (ref)							
**Currently working**							
Yes	0.75	0.51	1.10	0.58	0.34	0.96	0.035
No (ref)							
**Ever used the internet**							
Yes	0.91	0.73	1.13	0.75	0.55	1.03	0.076
No (ref)							
**Socioeconomic status**							
Lowest	1.21	0.94	1.56	1.38	0.96	1.99	0.079
Middle	1.69	1.32	2.17	1.34	0.98	1.85	0.071
Highest (ref)							
**Justifies wife-beating**							
Yes	1.25	0.98	1.61	1.25	0.97	1.62	0.091
No (ref)							
**Believe FGM is required by religion**							
Yes	1.08	0.87	1.34	0.90	0.71	1.14	0.389
No (ref)							
**Participates HH decision-making**							
Yes	0.72	0.60	0.86	0.78	0.64	0.96	0.016
No (ref)							
**Type of residence**							
Rural	0.81	0.64	1.03	0.98	0.71	1.34	0.916
Urban (ref)							
**Region**							
Somaliland	0.58	0.47	0.72	0.52	0.41	0.66	0.000
Somalia (ref)							

Note. OR = odds ratio, AOR = adjusted odds ratio, ref. = reference Category.

The results account for the 2020 SHDS complex sample design.

## Discussion

The study offered detailed analyses of the extent of early marriage among ever-married Somali women aged 20–49 and developed a set of determinants of the likelihood of marriage before 15 or 18 years old in Somaliland and Somalia, focusing specifically on socioeconomic determinants, geographic variation, and the role played by contextual determinants. The mean age of the women in the study was 31 years, with 64% residing in rural areas, a significant portion, 84% having no formal education, and 91% being unemployed. It is within this background that the prevalence of early marriage was investigated among the ever-married Somali women.

The research indicated a worrying percentage of early marriage, with 41.7% of women married before the age of 18 and 24.3% before the age of 15. This marks an alarming level for Somali women’s public health and human rights, as they are subject to such systematic discrimination. The study findings are also in line with data from other regions in Sub Saharan Africa and South Asia [42–44]. The study indicates that early marriage is more common among women aged 20–24 compared to women aged 40–49, showing that this practice continues to persist. A main reason for this high rate of early marriage among young women is probably the past thirty years of instability caused by war, extreme poverty, and political instability, which have drastically reshaped social norms [16]. Regionally, compared to women from Somaliland, Somali women had a higher rate of early marriage, possibly because of the variation in cultural practices and policy, in addition to the stability of Somaliland in contrast to Somalia’s decades of conflict and instability.

Significant differences by education and employment were not observed among women aged 20–49, probably due to the lack of education and employment was so pervasive that they failed to differentiate early marriage prevalence significantly. However, among women aged 20–29, significant effects of both education and work status were found in model three, though the education effect was in the opposite direction. A likely explanation of this unexpected finding is the fact that nearly all the women in the “primary or higher” education category were at the primary education level, which may not be sufficiently protective against early marriage. Women with low socioeconomic status had higher odds ratios of marrying before 18, which is consistent with the general global research, where poverty and fewer educational opportunities are associated with a higher incidence of early marriage [4,45]. Women who have ever used the internet had lower odds ratios of entering into early marriage, highlighting the role of digital exposure in delaying early marriage.

The finding that participation in household decision-making decreases the odds of early marriage is in line with [46] and suggests that women who have a say in household matters are likely to have more autonomy and empowerment. The increased autonomy enables women to delay marriage and pursue education or employment, reducing the chance of early marriage. Also, the findings show that the acceptance of domestic violence is intertwined with early marriage. This acceptance may portray a culture that perpetuates gender inequality.

These findings suggest that educational attainment alone in Somalia cannot prevent early marriage unless efforts are made to counteract cultural and socioeconomic determinants. Poverty needs to be alleviated. Improving women’s economic opportunities can prevent early marriage [47]. Empowering women within the household can postpone early marriage, with gender-norm-challenging programs being essential. Digital education and information are also powerful empowerment tools.

The findings highlight the need for a more holistic policy that addresses humanitarian issues as well as mitigating the rates of early marriage in Somalia and Somaliland. In particular, making it easier to fight poverty through economic access targeted towards women is fundamentally important for addressing the underlying economic drivers. Social and gender norms supporting family violence require active mitigation to enhance decision-making power of women at home, which increases their autonomy, and must be included in policies aimed at women’s empowerment. Additionally, policies on the internet as well as digital literacy for school-aged girls and women should be put into consideration due to the inverse relationship between the use of the internet and the rate of early marriages. Lastly, raising educational standards above primary level is absolutely crucial specially when basic education may not postpone early marriage. Collectively considering these areas requires collaboration across multiple sectors including health, education, economy, and social welfare to appropriately respond to the sophisticated context factors related to early marriage.

Though the study was based on a representative probability sample, the data was collected through personal interviews, an approach that is susceptible to either recall bias or social desirability bias, particularly for sensitive topics, such as the age at first marriage or attitudes toward intimate partner violence. The cultural aspects that drive early marriage cannot be fully understood using quantitative measures exclusively; qualitative research is also necessary for an in-depth understanding. Finally, mild age heaping indicates some irregularities in reporting age at first marriage among women. These limitations need to be taken into account cautiously while interpreting the results of this study.
